# ELISA-based assay of immunoglobulin G antibodies against mammalian cell entry 1A (Mce1A) protein: a novel diagnostic approach for leprosy

**DOI:** 10.1590/0074-02760160549

**Published:** 2017-12

**Authors:** Filipe R Lima, Iukary Takenami, Maurílio AL Cavalcanti, Lee W Riley, Sérgio Arruda

**Affiliations:** 1 Fundação Oswaldo Cruz-Fiocruz, Centro de Pesquisas Gonçalo Moniz, Laboratório Avançado de Saúde Pública, Salvador, BA, Brasil; 2 Escola Bahiana de Medicina e Saúde Pública, Salvador, BA, Brasil; 3 Hospital Couto Maia, Secretaria de Saúde do Estado da Bahia, Salvador, BA, Brasil; 4 University of California, School of Public Health, Division of Infectious Diseases and Vaccinology, Berkeley, CA, USA

**Keywords:** leprosy, antibodies, Mce1A protein, diagnosis, immunoglobulin

## Abstract

**BACKGROUND:**

Leprosy is a chronic infectious disease caused by the obligate intracellular bacillus *Mycobacterium leprae.* Because leprosy diagnosis is complex and requires professional expertise, new tools and methodologies are needed to detect cases in early stages and prevent transmission. The *M. leprae* genome contains *mce1A,* which encodes a putative mammalian cell entry protein (Mce1A). We hypothesised that the presence of Mce1A on the cell surface could be detected by the host's immune system.

**OBJECTIVE:**

The aim of this study was to evaluate antibody responses against the Mce1A protein in leprosy patients, household contacts of patients, and the general population to present an addition tool for leprosy diagnosis.

**METHODS:**

A cross-sectional study involving 89 volunteers [55 leprosy cases, 12 household contacts (HHC) and 22 endemic controls (EC)] was conducted at Couto Maia Hospital, in Salvador, Bahia (BA), Brazil.

**RESULTS:**

The median anti-Mce1A IgA was significantly higher in multibacillary (MB) and paucibacillary (PB) cases than in EC (p < 0.0001). A similar trend was observed in IgM levels, which were significantly higher in both MB (p < 0.0001) and PB (p = 0.0006) groups compared to in EC individuals. The greatest differences were observed for IgG class-specific antibodies against Mce1A. The median levels of MB and PB were significantly higher compared to both controls HHC and EC (MB or PB vs EC, MB vs HHC p < 0.0001; PB vs HHC, p = 0.0013). Among leprosy cases, IgG enzyme-linked immunosorbent assay sensitivity and specificity were 92.7% and 97.1%, respectively. IgG positivity was confirmed in 92.1% and 94.1% of MB and PB patients, respectively.

**CONCLUSION:**

This novel diagnostic approach presents an easy, non-invasive, and inexpensive method for leprosy screening, which may be applicable in endemic areas.

Leprosy, a disabling chronic infectious disease, is caused by the obligate intracellular bacillus *Mycobacterium leprae* and remains a public health problem in endemic areas of developing countries, such as Brazil (Rodrigues & Lockwood 2011). *M. leprae* tropism in reticuloendothelial and Schwann cells results in infection, mainly affecting the skin, peripheral nerves, mucosa of the upper respiratory tract and eyes (Eichelmann et al. 2013, WHO 2016). Skin lesions, neuropathological involvement resulting in loss of peripheral motor function, and deformities are historically responsible for the social stigma associated with this disease (Eichelmann et al. 2013).

According to official reports from 121 countries, the global registered prevalence of leprosy was 213,899 in 2014. This number of new cases indicates the high rate of continued transmission of infection by *M. leprae* (WHO 2016). The nasal mucosa is the most common route of transmission of the bacillus during close and frequent contact with leprosy patients (Martins et al. 2010). Accordingly, the early detection of disease spread and effective treatment with standard multidrug therapy is an important strategy for controlling leprosy (Rodrigues & Lockwood 2011). However, the WHO global strategy for reducing the leprosy burden may be unsuccessful without improved diagnostic tools.

Diagnosing leprosy is difficult and professional expertise is required to differentiate this disease from other skin diseases, including vitiligo, hypochromic eczematides, tinea corporis, and pityriasis veriscolor (Yang et al. 2013). The diagnosis of leprosy is based on clinical examination, bacilloscopy of the dermal lymph, and histopathology of skin lesion biopsies (WHO 2016). Although bacilloscopy and histopathology are highly specific, these techniques show low sensitivity (Amorim et al. 2016). Moreover, negative results do not exclude the diagnosis of leprosy (MS 2002). In addition, histopathology presents technical and practical limitations such as the invasive nature of this method and need for specific laboratory equipment.

*M. leprae* has been shown to reprogram macrophage differentiation, contributing to persistence of the bacillus in the host (Batista-Silva et al. 2016). Although modulation of immune mechanisms by mycobacteria have been comprehensively studied (Koul et al. 2004), we hypothesised that after infection, *M. leprae* facilitates changes in its environment to survive in the host by modifying the antigens in the mycobacterial wall. Proteins found in the bacillary cell wall are the first to interact with host immune cells; i.e., these proteins may stimulate the host immune system to evoke a cellular or humoral response (Mazini et al. 2016).

Mammalian cell entry 1A (Mce1A) (ML2589; mce1A) protein on the surface of *M. leprae* (Santhosh et al. 2005) was first identified in *Mycobacterium tuberculosis* by Arruda et al. (1993). Recombinant *Escherichia coli* expressing the *M. tuberculosis* Mce1A protein could invade and survive in HeLa cells and macrophages (Arruda et al. 1993). Although *M. leprae* was originally identified in *M. tuberculosis*, orthologs of mce genes are widely distributed throughout the *Mycobacterium* genus and are found in *M. leprae* (Haile et al. 2002). Sato et al. (2007) demonstrated that the *mce1A* gene product can mediate entry of *M. leprae* into epithelial cells of the respiratory tract, and that using anti-Mce1A antibodies in latex beads does not permit bacterial internalisation by epithelial cells.

The potential role of Mce1A protein in the invasion of host cells suggests that this protein, which may interact with immune defense cells, represents a target for the development of novel diagnostic approaches. Accordingly, the aim of this study was to evaluate serum antibody responses against Mce1A protein in patients diagnosed with leprosy to determine whether this response can be used as a biomarker to aid in the early diagnosis of leprosy.

## MATERIALS AND METHODS

*Setting* – A cross-sectional study was conducted at Couto Maia Hospital (HCM), a reference unit for the treatment of leprosy patients in Salvador, Bahia (BA), Brazil. Volunteer subjects were recruited using convenience sampling between June 2014 and December 2015.

*Study population* – The present study was approved by the Institutional Review Board for Human Research of the Oswaldo Cruz Foundation in Salvador. After signing an informed consent form, volunteers were classified into three groups: leprosy cases, household contacts (HHC), and endemic controls (EC) (Table I).

**TABLE I:** Study population characteristics (N = 89)

Variable		EC (n = 22)	HHC (n = 12)	Leprosy (n = 55)	p-value
Age, years		28 (21-49.3)	31 (29-38)	42 (30.8-53.3)	0.043*^a^*
Sex, n (%)					
	Male	6 (33.3)*^c^*	2 (18.2)*^d^*	24 (43.6)	0.254*^b^*
	Female	12 (66.7)	9 (81.8)	31 (56.4)	
Cases, n (%)					
	PB	–	–	17 (30.9)	–
	MB	–	–	38 (69.1)	
Classification, n (%)					
	ID	–	–	6 (10.9)	–
	TT	–	–	9 (16.4)	
	BT	–	–	3 (5.5)	
	BB	–	–	4 (7.3)	
	BL	–	–	22 (40)	
	LL	–	–	11 (20)	

Age and skin lesions shown as median values (IQR). EC: endemic control; HHC: household contacts of leprosy patients; PB: paucibacillary; MB: multibacillary; ID: indeterminate; TT: tuberculoid; BT: borderline-tuberculoid; BB: borderline-borderline; BL: borderline-lepromatous; LL: lepromatous.

a comparison of three groups using the Kruskal-Wallis Test;

b comparison of three groups using Chi-Squared;

c data not available for four volunteers;

d data not available for one volunteer.

*Leprosy cases* – Newly diagnosed leprosy patients older than 18 years of age seen at HCM were invited to participate in this study. According to the WHO and Brazilian Ministry of Health, patients were considered eligible for inclusion in the study if their diagnosis was confirmed by clinical evaluation, bacilloscopy and/or biopsy (MS 2010, WHO 2016). Skin smears were taken from four sites (both earlobes and elbows) and processed using the Ziehl-Neelsen staining technique to detect acid-fast bacilli by microscopy. Acid-fast bacilli were graded using a bacillary index (BI) according to the Ridley scale (0-6+) (MS 2010) and the average score of individual smears was recorded. In addition, skin biopsies were taken and examined by an anatomical pathologist. The patients were then classified according to the Ridley-Jopling criteria: indeterminate leprosy (ID), tuberculoid leprosy (TT), borderline tuberculoid (BT), borderline borderline (BB), borderline lepromatous leprosy (BL), and lepromatous leprosy (LL). For treatment purposes, patients were stratified as either paucibacillary (PB), considered as up to five skin lesions and/or a negative BI, or multibacillary (MB) when they had more than five lesions and/or positive BI.

*Household contacts* – HHC were defined as adults residing in the same household with an index case for at least six months prior to diagnosis. All HHC and EC were clinically screened for signs or symptoms suggestive of leprosy. Clinical examinations were performed by trained physicians and health professionals at HCM.

*Endemic controls* – EC, representing community contacts, were defined as individuals residing in an endemic area who had no history of contact with a leprosy patient.

All participants reported being human immunodeficiency virus-negative and did not use immunosuppressive drugs. Prior to inclusion, all participants were assessed with respect to latent tuberculosis infection with the QuantiFERON^®^ TB Gold In-Tube test (QFT-IT; Cellestis Limited, Carnegie, Victoria, Australia).

*Samples and enzyme-linked immunosorbent assay (ELISA)* – Blood was collected by venipuncture and serum samples were stored at −20° C until use. Quantitative assessment of IgA, IgM and IgG antibodies against the Mce1A protein was performed by indirect ELISA (Takenami et al. 2016). Purified recombinant Mce1A protein was provided by Dr LW Riley (University of California, Berkeley, CA, USA). Mce1A protein (10 μg/mL) was diluted to 1:1000 in ethanol and 50 μL of this solution was dried overnight on polystyrene ELISA well plates (Greiner bio-one, Kremsmunster, Austria). ELISA plates were then blocked with 100 μL of 3% low fatty-acid bovine serum albumin (BSA) (US Biologicals, Salem, MA, USA) and washed with phosphate-buffered saline (PBS, pH 7.4) (GIBCO, Grand Island, NY, USA). Frozen serum samples were thawed and diluted 1:100 in 3% BSA. Next, 100 μL of each diluted sample was added to plates and incubated for 1 h at room temperature (RT) between 18-25°C, followed by three washes with 1x PBS. Next, 100 μL of 1:10.000, 1:50.000, or 1:10.000 of goat-derived anti-human IgM, IgG, or IgA, respectively, labeled with horseradish peroxidase (Sigma-Aldrich, St. Louis, MO, USA) diluted in 3% BsA/PBS, was added, followed by incubation at RT for 1 h. This was followed by repeated washing with 1x PBS. The secondary antibodies were tested by titration to determine the optimum working dilution. Tetramethylbenzidine (100 μL) solution (Invitrogen Life Technologies, Carlsbad, CA, USA) was added and the plates were reincubated for 1 h at RT. Finally, the reaction was stopped with 100 μL of 2N sulfuric acid. Reactions were read within 10 min at 450 nm in a spectrophotometer (Thermo Scientific, Waltham, MA, USA). Results were recorded as the average of optical density of triplicate samples, and the assay was repeated if the coefficient of variance was > 10%.

*Statistical analysis* – All data were analysed by GraphPad Prism v.7.0 software (GraphPad Inc., La Jolla, CA, USA). Statistical variations were analysed by the Kruskal-Wallis test, followed Dunn's test. Spearman's correlation was used to compare immunoglobulin levels and BI or skin lesions. The ability of immunoglobulin levels to discriminate leprosy patients from controls (EC or HHC) was evaluated using receiver operating characteristic (ROC) curves. Chi-square test was used to assess associations among categorical variables. The level of statistical significance was set at p < 0.05.

## RESULTS

**Fig. 1:**
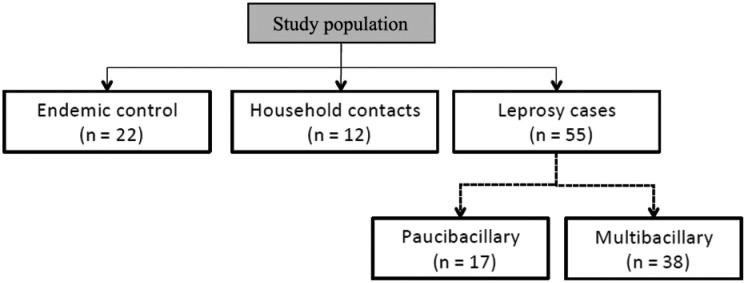
flowchart of study population selection process (n = 89).

*Study population characteristics* – The present study included 89 volunteer subjects, grouped as leprosy cases (n = 55; 61.8%), HHC (n = 12; 13.5%), and EC (n = 22; 24.7%) (Fig. 1). Of the 55 leprosy cases, 17 (30.9%) were PB and 38 (69.1%) were MB. The PB patient group included nine (52.9%) patients classified as TT, six (35.3%) as ID, one (5.9%) as BT, and one (5.9%) as BL. BL patients were classified as PB by the number and size of lesions (less than five lesions) and dermatological and histopathological characteristics. In addition, both showed negative smear microscopy results. The MB patient group was comprised of 11 (28.9%) LL, 21 (55.3%) BL, four (10.5%) BB, and two (5.3%) BT.

Bacillus Calmette-Guérin (BCG) scars were detected in 70.6% (12/17) of PB leprosy patients and 65.8% (25/38) of MB patients. Descriptive characteristics of the study population are summarised in Table I. The familial relationships of HHC with leprosy patients were spouses (25%), sons/daughters (25%), mothers (33.4%), fathers (8.3%), and daughters-in-law (8.3%). A significant difference was observed between the ages of leprosy patients and control individuals (p = 0.043). The prevalence of QFT-IT positivity in leprosy cases, HHC, and EC was 9.1%, 25.0%, and 14.4% respectively, but the differences were significant (p = 0.309).

**Fig. 2:**
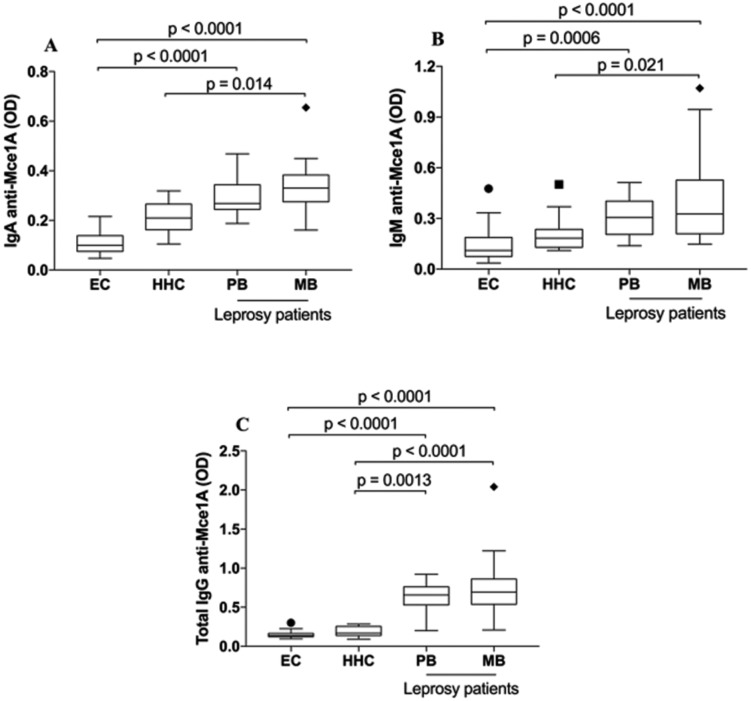
comparison of IgA (A), IgM (B), and IgG (C) antibody levels against Mce1A protein among endemic controls (EC) (n = 22), household contacts (HHC) (n = 12), and leprosy cases (n = 55). Serum levels were analysed using the Kruskal-Wallis test, followed by the post-hoc Dunn test. PB: paucibacillary; MB: multibacillary.

*Anti-Mce1A antibody levels* – The antibody profiles against Mce1A protein in leprosy cases, HHC, and EC are shown in Fig. 2. All levels of immunoglobulins were significantly higher in leprosy cases than in the control groups (p < 0.0001). The median values of IgA against Mce1A protein were significantly higher in MB [median: 0.330 (IQR: 0.274-0.382), p < 0.0001] and PB [median: 0.268 (IQR: 0.244-0.343), p < 0.0001] cases than in EC [median: 0.099 (IQR: 0.075-0.138)] (Fig. 2A). In addition, MB cases presented a higher median optical density than did HHC individuals [median: 0.209 (IQR: 0.162-0.265), p = 0.014].

A similar trend was observed with respect to IgM levels, which were significantly higher in both the MB [median: 0.326 (IQR: 0.209-0.527), p < 0.0001] and PB [median: 0.305 (IQR: 0.205-0.420), p = 0.0006] groups than in EC individuals [median: 0.110 (IQR: 0.075-0.187)]. In contrast, only MB cases showed significant differences in IgM levels compared to HHC cases [median: 0.183 (IQR: 0.129-0.235), p = 0.021] (Fig. 2B). The greatest differences were observed in IgG class-specific antibodies against Mce1A. The median levels of MB [median: 0.695 (IQR: 0.538-0.860)] and PB [median: 0.655 (IQR: 0.529-0.760)] were significantly higher compared to both controls [HHC, median: 0.168 (IQR: 0.137-0.254), p < 0.0001; EC, median: 0.138 (IQR: 0.1240.166), p < 0.0001 and p < 0.013, respectively] (Fig. 2C).

**Fig. 3:**
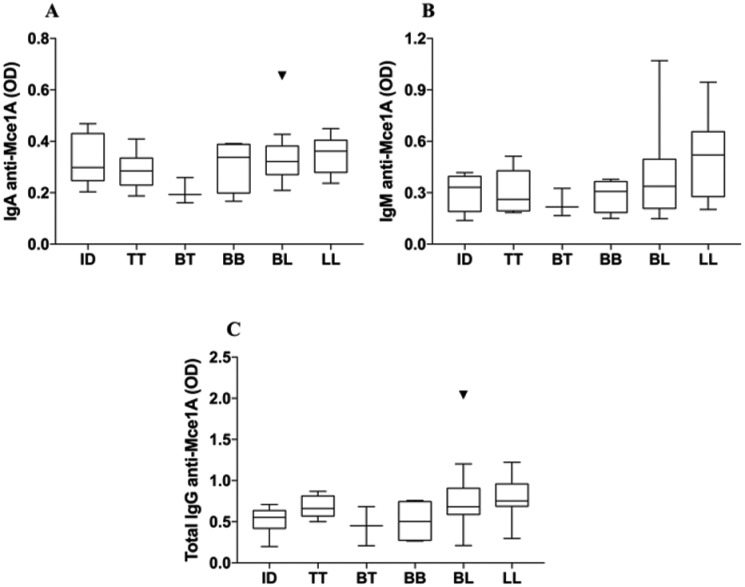
levels of IgA (A), IgM (B), and IgG (C) anti-Mce1A in leprosy patients grouped by Ridley-Jopling classification. Serum levels were analysed using the Kruskal-Wallis test followed by the post-hoc Dunn test. Indeterminate (ID) (n = 6); tuberculoid (TT) (n = 9); borderline- tuberculoid (BT) (n = 3); borderline-borderline (BB) (n = 4); border- line-lepromatous (BL) (n = 22); lepromatous (LL) (n = 11).

*Correlations between antibody titers and clinical data* – Leprosy patients showed a high degree of variability in anti-Mce1A antibody levels when samples were stratified according to Ridley and Jopling (1966) criteria (p > 0.05 all immunoglobulins) (Fig. 3). A weak correlation was observed between anti-Mce1A IgM and IgG titers and BI. In contrast, BI showed a positive correlation with the levels of IgA (Table II). Similarly, IgA and IgM antibody levels were positively correlated with the number of skin lesions (Table II). As expected, the number of skin lesions was associated with Mb classification.

**TABLE II:** Correlations between serum immunoglobulin levels, bacillary index and skin lesions

Variables	Correlation coefficient (R)	p-value
BI vs. IgA	0.291	0.043
BI vs. IgM	0.135	0.354
BI vs. IgG	0.070	0.633
Skin lesions vs. IgA	0.275	0.042
Skin lesions vs. IgM	0.316	0.019
Skin lesions vs. IgG	0.121	0.380

The correlation coefficient was analysed using Spearman's test. BI: bacillary index.

**Fig. 4:**
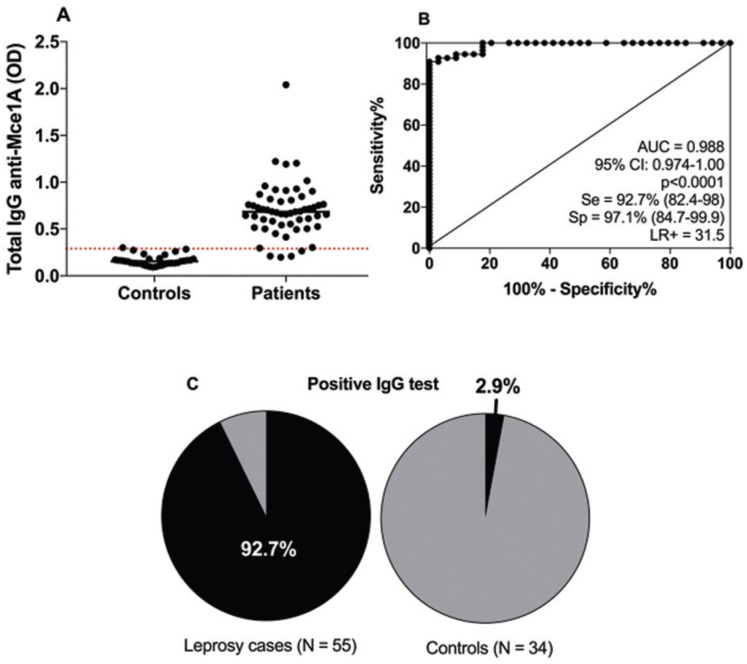
levels of IgG anti-Mce1A in serum of leprosy patients and controls (A). Receiver operating characteristic (ROC) analysis was used to distinguish leprosy patients from endemic controls (EC) and household contacts (HHC) (B). Positivity to IgG among leprosy patients and control groups. Pairwise comparisons of proportions were performed using Fisher's test, p < 0.0001 (C). AUC: area under the curve; CI: confidence interval; Se: sensitivity; Sp: specificity; LR: likelihood ratio.

*Antibodies against Mce1A in leprosy cases* – We performed ROC analysis for all immunoglobulin ELISA results (data no shown). However, the best overall performance was observed for the IgG antibody. The area under the ROC curve for anti-Mce1A IgG antibody levels in leprosy patients versus controls (EC and HHC) was 0.988 (p < 0.0001), with 92.7% sensitivity and 97.1% specificity (Fig. 4A-B). Anti-Mce1A IgG was positive in 92.7% (51/54) of leprosy patients and 2.9% (1/34) of controls. Among the Mb and PB patients, 92.1% and 94.1% were positive for anti-Mce1A IgG, respectively. Significant differences in positivity rates were observed between leprosy patients and control individuals (Fig. 4C).

## DISCUSSION

Previous studies have suggested that the true prevalence of leprosy is underestimated because of diagnostic limitations inherent to large-scale control programs aimed at eventual disease elimination (Amorim et al. 2016, WHO 2016). Thus, we evaluated the potential of a Mce1A-dependent antibody response against *M. leprae* as an alternative method for diagnosing leprosy.

The tuberculoid form of this disease may exhibit a strong cell-mediated immune response, and gene expression profiles are associated with inflammation, leading to the control of bacillary multiplication (Belone et al. 2015). In the lepromatous form, patients exhibit a defective cell-mediated immune response to *M. leprae,* resulting in a high bacillary load (Batista-Silva et al. 2016). However, we observed that in all clinical forms of leprosy, the Mce1A protein activates the humoral immune response, inducing production of high levels of antibodies against this protein. However, these increased serum antibody concentrations were unable to block the multiplication of *M. leprae* in the host. Thus, our results are consistent with those of other studies suggesting that antibody production is detectable and, as such, is a potential serologic biomarker, despite the absence of protective effects in controlling mycobacterial load (Nath et al. 2015). Moreover, it is possible that the clinical forms, pathological manifestations, and association with increased susceptibility are determined by regulatory T cells (CD4+CD25+FOXP3+), which suppress the effetor T cell function elicited by the host during intracellular infection by *M. leprae* (Bobosha et al. 2014, Holla et al. 2016).

Although antibody levels cannot be used to differentiate the clinical forms of this disease, antibody production against Mce1A allows us to distinguish leprosy cases from controls, EC and/or HHC. Immunoglobulin G showed the best diagnostic performance, with sensitivity and specificity of 92.7% and 97.1%, respectively. Furthermore, seropositivity among leprosy cases in this study was higher (IgG: 92,7%) than that against the response of a single chimeric protein with leprosy IDRI diagnostic (LID)-1 and phenolic glycolipid (PGL)-I epitopes (64%) found by Amorim et al. (2016), designated as LID-NDO ELISA, and 74.4% by Duthie et al. (2014). Overall the results from studies using PGL-I showed that the average seropositivity range was 23% and 78% in the PB and MB groups, respectively, and these values vary depending on the antigen preparations and ELISA protocols used (de Moura et al. 2008). A study by Mizoguti et al. (2015) showed that the rate of seropositivity among non-reactional MB patients was 75.0% for anti-LID-1 and 67.0% for anti-PGL-I antibodies. However, the performance of anti- Mce1A was not compared to those obtained using well- known and well-established standards using other antigens. Additionally, no studies have examined anti-Mce1A antibodies in leprosy patients, and thus it is difficult to compare our results with those of other studies.

In the control group, only one (2.9%) volunteer showed positive results in IgG ELISA. Interestingly, the only positive volunteer for the IgG test was reported to work with *Mycobacterium avium* for a long period. Parker et al. (1995) showed the presence of a similar gene in *M. avium* and, therefore, previous infection with that species may have contributed to the positive result. Although no significant correlations with BI and the number of skin lesions were detected, IgG against Mce1A enabled identification of patients with PB (94.1%) and MB (92.1%) leprosy. Accordingly, the antibody response appeared to be independent of bacillary load and clinical classification. In contrast, both IgA and IgM showed a positive linear correlation between immunoglobulin levels and BI, as well as the number of skin lesions. These findings suggest that the Mce1A protein can induce immunoglobulin class switching via an unknown mechanism.

Elevated antibody levels against Mce1A were also detected in patients with tuberculosis (TB). However, median antibody levels were significantly higher in TB patients compared to in leprosy cases. Because TB patients present a wide range of IgG levels against Mce1A (Takenami et al. 2016), we hypothesised that the serum of individuals infected by *M. tuberculosis* is responsible for eliciting cross-reacting antibodies which, consequently, led to the increased antibody levels in coinfected leprosy cases. However, all volunteers were screened for latent TB infection using QFT-IT rather than tuberculin skin test because most volunteers were vaccinated for BCG (79.8%), and no differences were detected among the three groups (p > 0.05). In addition, no significant differences were found between positive and negative QFT-IT results (p > 0.05). Despite differences in Mce1A protein gene encoding between *M. leprae* and *M. tuberculosis*, this protein appears to play a similar role in enabling of bacillary invasion into mammalian cells (Santhosh et al. 2005). Little is known about the role of this protein in leprosy patients. Additional studies are needed to understand the homology between mce1A genes of *M. tuberculosis* and *M. leprae* and its potential implications for new strategies of leprosy diagnosis.

The production of anti-Mce1A antibodies (IgG, IgA, and IgM) was not significant (p > 0.05) among PB and MB volunteers, suggesting that leprosy can be detected over a disease spectrum by a screening serological test. The identification of protein antigens with biomarker potential may contribute to the development of a serologic assay for improving leprosy case detection (Hungria et al. 2012). Laboratory evaluations of skin smears in this study were positive in only 11 (20%) patients, while 41 (74.5%) and three (5.5%) were negative and not performed, respectively (data not shown). Negative skin smear results were observed regardless of clinical form or disease severity, demonstrating the inadequate capacity of skin smears to accurately detect leprosy, regardless of clinical classification, as only 17 (30.9%) of the negative results were classified as PB. In contrast, serum IgG antibody levels were used to confirm 38 (92.7%) of the negative skin smear results.

The present study was limited by the small number of volunteers, and further studies of a larger sample and anti-PGL-1 serology are needed to provide more definitive estimates of accuracy. For operational reasons, an anti-PGL-1 test were not performed. However, cross-reactivity has not been thoroughly evaluated, restricting the use of this test. Additional studies are necessary to evaluate the performance of serological assays in a cohort LTBI and active TB patients and other relevant skin diseases must be investigated, such as pityriasis versicolor and vitiligo, which may be clinically confounded with leprosy.

Our results suggest that detection of IgG antibodies against Mce1A is highly specific and sensitive for detecting leprosy cases in an endemic region. Accordingly, IgG ELISA against Mce1A may be useful as an easy, non-invasive, and inexpensive diagnostic method of leprosy screening. Further studies are required to more comprehensively understand the role of this protein in the pathogenesis of *M. leprae.* A parallel study is currently being conducted to more comprehensively follow HHC of leprosy patients to monitor IgG antibody responses to Mce1A protein to provide insight into early diagnosis prior to the appearance of lesions.
